# Sustained Cortical and Subcortical Measures of Auditory and Visual Plasticity following Short-Term Perceptual Learning

**DOI:** 10.1371/journal.pone.0168858

**Published:** 2017-01-20

**Authors:** Bonnie K. Lau, Dorea R. Ruggles, Sucharit Katyal, Stephen A. Engel, Andrew J. Oxenham

**Affiliations:** Department of Psychology, University of Minnesota, Minneapolis, Minnesota, United States of America; Harvard Medical School, UNITED STATES

## Abstract

Short-term training can lead to improvements in behavioral discrimination of auditory and visual stimuli, as well as enhanced EEG responses to those stimuli. In the auditory domain, fluency with tonal languages and musical training has been associated with long-term cortical and subcortical plasticity, but less is known about the effects of shorter-term training. This study combined electroencephalography (EEG) and behavioral measures to investigate short-term learning and neural plasticity in both auditory and visual domains. Forty adult participants were divided into four groups. Three groups trained on one of three tasks, involving discrimination of auditory fundamental frequency (F0), auditory amplitude modulation rate (AM), or visual orientation (VIS). The fourth (control) group received no training. Pre- and post-training tests, as well as retention tests 30 days after training, involved behavioral discrimination thresholds, steady-state visually evoked potentials (SSVEP) to the flicker frequencies of visual stimuli, and auditory envelope-following responses simultaneously evoked and measured in response to rapid stimulus F0 (EFR), thought to reflect subcortical generators, and slow amplitude modulation (ASSR), thought to reflect cortical generators. Enhancement of the ASSR was observed in both auditory-trained groups, not specific to the AM-trained group, whereas enhancement of the SSVEP was found only in the visually-trained group. No evidence was found for changes in the EFR. The results suggest that some aspects of neural plasticity can develop rapidly and may generalize across tasks but not across modalities. Behaviorally, the pattern of learning was complex, with significant cross-task and cross-modal learning effects.

## Introduction

One of the remarkable feats of perceptual neural processing is the ability to learn, change, and adapt to particular circumstances and tasks. Auditory plasticity has been demonstrated on multiple time scales and in a wide variety of contexts. Directed attention very quickly enhances the cortical representation of relevant sounds [1], and even very brief (1–5 s) listening experience in reverberant [2,3] or spectrally colored [4,5] environments can improve speech intelligibility. Long-term experience, such as musical training and tonal language fluency, have been associated with neural plasticity that may develop over the course of years [6–8]. The physiological mechanisms that underlie long- and short-term plasticity in auditory perceptual processing continue to be elusive but have significant implications for understanding the neurophysiology of the auditory pathways and for developing interventions for clinical populations. Similar questions of mechanism and time scale are of interest in the visual domain [9].

Long-term musical training has been extensively studied in terms of its impact on auditory perception as well as the encoding of sound along the auditory pathway. Although it is difficult to rule out genetic and social factors that may influence neural development as well as the pursuit of musical training, both cortical and subcortical neurophysiological enhancements have been associated with musical experience. Structural neuroimaging studies report increased gray matter volume in auditory, motor, and visuo-spatial cortical regions in professional musicians [8,10,11]. Enhancement of cortical evoked potentials including N1 and P2 [12,13] and the earlier N19m-P30m complex [10] have been observed in response to tonal stimuli. Sustained responses to periodic stimuli of 80–1000 Hz (EFR) are thought to be generated primarily in the subcortical auditory structures [14,15], and investigations of these responses have shown greater spectral magnitude of the fundamental frequency (F0; the acoustic correlate of pitch) of complex tones in musicians compared to non-musicians [16]. Behavioral results are consistent with these physiological findings, with superior frequency-discrimination abilities reliably reported in musicians compared to non-musicians [17,18]. However, whether such auditory perceptual enhancements generalize beyond music-related tasks, such as frequency discrimination is unclear [19]. For instance, improvements in speech perception in noise or interfering talkers have been reported by some groups [20–22] but not by others [23–26]. Improved understanding of plasticity in EFR after shorter, more controlled learning may help to clarify details of the subcortical plasticity attributed to musical training.

Studies of learning-induced plasticity in non-human primates and rats have shown expanded representation of trained frequencies in primary auditory cortex following frequency-discrimination training [27–29]. Much less is known about the cortical correlates of short-term perceptual learning in humans, but several studies report enhancement of the N1 and P2 cortical event-related potentials (ERP) following auditory training [30,31]. In this study, we investigate whether plasticity can also be observed in sustained steady-state EEG responses, which reflect the entrainment of neural responses to the periodicity of external stimuli, as opposed to previous time-domain ERP measures. We paired three tasks involving auditory or visual discrimination with corresponding steady-state responses. All behavioral tasks required discrimination as opposed to detection, in order to target high-level perceptual judgments [32].

Pitch discrimination can be quickly learned, to the extent that initially untrained listeners can achieve the same level of performance as professional musicians with 4–6 hours of training [33]. Pitch discrimination also offers the opportunity to study short-term perceptual learning using a task that is also often associated with long-term plasticity. We trained listeners on pitch discrimination with harmonic complex tones, which elicit an EFR in response to the F0. The EFR is a broadly generated response which has been localized primarily to subcortical neural populations, and which reflects phase-locked responses to amplitude modulation in the range of 80–1000 Hz [14,15,34]. Although reports of plasticity following short-term perceptual learning are mainly limited to cortical measures, at least one study investigated the possibility of subcortical plasticity in sustained responses [35]. In their study, Carcagno and Plack [35] found significant enhancement of EFR amplitude in response to dynamic and static pitch stimuli, along with improvements in behavioral discrimination. However, their results were inconsistent for the dynamic conditions and the effects were sufficiently small to warrant replication.

The processing of AM is central to speech perception in quiet and noise [36–38], with modulation rates below about 16 Hz most important for speech understanding in general [39,40]. AM rate discrimination also improves with training and shows minimal generalization to other tasks, making it ideal for combining with frequency discrimination to study specificity of learning and plasticity along the auditory pathway [41]. We tested AM rate discrimination training with amplitude-modulated complex tones, which also elicit an auditory steady state response (ASSR). The ASSR in response to AM between 0–40 Hz is thought to be generated in the auditory cortex [15].

The third set of measures included here involves a visual perceptual training task combined with steady-state visual evoked potentials (SSVEP). Visual and auditory steady-state responses are similar but independently generated, allowing us to investigate potential cross-modal transfer effects. Discrimination of Gabor pattern orientation improves with training [42] and some evidence suggests that the SSVEP is plastic in response to both perceptual training and aversive conditioning [43,44].

The aim of this study was to evaluate the influence of perceptual learning on sustained neurophysiological responses within and across sensory modalities. Each auditory stimulus had both an F0 and an AM rate, allowing us to test whether training on one feature (e.g., F0) affected the neural representation and perception of another feature (e.g., AM). Concurrent presentation of the AM and F0 also enables simultaneous measurements from potentially different auditory neural generators. Our hypothesis was that F0-discrimination training would selectively enhance the EFR to the F0, whereas AM-discrimination training would selectively enhance the ASSR to the AM rate, and that the visual orientation discrimination training would selectively enhance the SSVEP responses.

## Materials & Methods

### Subjects

The participants were forty (25 females and 15 males) normal-hearing listeners between 18 and 35 years of age who had less than 5 years of musical training, no prior experience in psychophysical experiments, and who did not speak any tonal languages. All participants passed an audiometric screening with pure-tone thresholds below 20 dB hearing level (HL) for octave frequencies between 250 and 8000 Hz and wore visual corrective lenses as prescribed by an optometrist during all sessions if required. Written informed consent was obtained from all participants in accordance with protocols reviewed and approved by the Institutional Review Board at the University of Minnesota. The participants were paid for their participation.

### Stimuli

**Fig 1:**
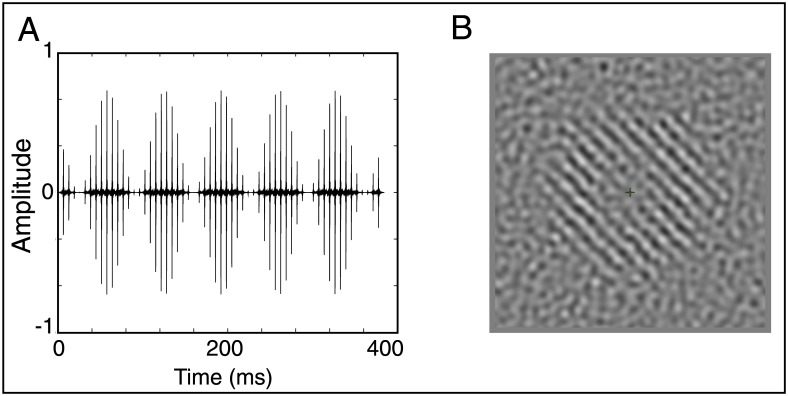
Auditory and visual stimuli. (A) Unresolved harmonic complex with a F0 of 137 Hz, amplitude modulated at 13 Hz. (B) 3° of visual angle standard deviation degree Gabor pattern with spatial frequency of 1 degree per cycle embedded in a 9° background noise.

The auditory stimuli were amplitude-modulated harmonic complexes (Fig 1A) with all components added in sine phase, bandpass filtered between 1700 and 3600 Hz (Butterworth filter with 24 dB/oct slopes). This filtering ensured that only harmonics 17 to 33 were presented, which in turn meant that the participants had to rely on the periodicity in the temporal envelope to extract the pitch [45,46]. In this way, both the perception and the EFR relied on the same acoustic cues. The nominal F0 of the complex was 137 Hz, and the complex was sinusoidally amplitude modulated at a rate of 13 Hz with 100% depth. The duration of the complexes was 400 ms for behavioral tasks and 1 s for the EEG measurements, and all complexes had 10-ms raised-cosine onset and offset ramps. Modulated tones were embedded in a threshold equalizing noise (TEN) [47] extending from 50 to 8000 Hz, with a spectral notch from 1500 to 4800 Hz to limit the contributions from neurons tuning to frequencies outside the stimulus passband, and to reduce the audibility of any distortion products.

During behavioral testing, the tones were presented at an overall level of 53 dB sound pressure level (SPL), and the TEN level was 43 dB SPL per equivalent rectangular bandwidth (ERB) at 1 kHz between 50 and 1500 Hz and 33 dB SPL per ERB between 4800 and 8000 Hz. During EEG recording, the tones were presented at an overall level of 65 dB SPL embedded in TEN at 55 dB SPL in the lower region and 45 dB SPL in the higher region. The tones were increased by 10 dB during EEG recording because higher presentation levels (65–80 dB SPL) recruit larger neural populations and maximize the signal-to-noise ratio in recorded potentials [34,35,48].

The visual stimuli were generated using Psychophysics toolbox [49–51] in MATLAB (The MathWorks Inc., Natick, MA). The visual stimuli consisted of a 3° of visual angle standard deviation Gabor pattern with a spatial frequency of 1 cycle per degree embedded in a 9° background of filtered noise (Fig 1B). The noise was created by convolving white noise with an isotropic 2D Gaussian filter centered on 1 cycle per degree with a standard deviation of 0.33 cyc per degree. In order to record the SSVEP to both the stimuli and the background noise, the Gabor pattern sawtooth flickered at 13 Hz while the noise sawtooth flickered at 17 Hz.

### Protocol

The perceptual training paradigm was conducted with four groups of ten participants, assigned randomly to each group. In the three experimental groups, participants were trained for about 30 minutes a day for 6 days on one of three tasks: F0 discrimination (F0 Group), AM rate discrimination (AM Group), or visual orientation discrimination (VIS Group). Participants were encouraged to complete the sessions on consecutive days but were allowed a maximum of two days between training sessions if they were unable to attend a weekend session. The final training session and the post-test were always completed on consecutive days. The EEG recording and behavioral measures were repeated again 30 days post-training to investigate whether training effects were maintained. A fourth, no-training control group (CON Group) was also included, where participants received EEG recording and behavioral pre- and post-tests at comparable time intervals to participants in the other groups but were not trained on any discrimination task. Control group participants received the initial EEG and behavioral pre-test and returned one week later to complete the post-test but did not participate in the maintenance test.

### EEG test procedure

Pre-, post- and maintenance tests consisted of an EEG recording session followed by behavioral threshold measurements for each of the three discrimination tasks. EEG measurements were acquired using a Biosemi active electrode system with a sampling rate of 4096 Hz and 32 channels referenced to averaged mastoid electrodes. The recordings were made in a sound-attenuating booth.

The SSVEPs were recorded in response to a Gabor pattern embedded in background noise (Fig 1B), partitioned into twenty 1-minute blocks. Participants were seated two feet in front of an HP LP2065 LCD monitor with a refresh rate of 75 Hz. The monitor’s luminance gamma curves were measured using a Photoresearch PR-655 and corrected in software to ensure correct display of stimulus intensity. During recording, participants were presented with a luminance-change discrimination task to ensure that they were attending to the stimuli. Within each 1-minute block, the intensity of the Gabor pattern increased ten times at random intervals. Participants were instructed to press a key on a number pad as quickly as possible whenever they detected the luminance change. All participants achieved 80% correct or better on the task. The blocks were self-paced and completed in less than 30 minutes for all participants.

Following the SSVEP, simultaneous cortical and subcortical auditory sustained responses were recorded to 1000 repetitions of the 1-s AM harmonic complex (Fig 1A). The (cortical) ASSR was measured to the 13-Hz AM rate while the (subcortical) EFR was measured to the 137-Hz F0 in the same tones. The complexes were generated using Matlab and played to participants via a Tucker Davis Technologies (TDT) real time processor with headphone buffer and Etymotic ER1 insert earphones. Interstimulus intervals were jittered randomly between 700 and 800 ms and stimulus polarity was randomly alternated, resulting in 500 presentations of each polarity. Participants watched a silent close-captioned movie while listening to the stimuli during the auditory EEG recordings.

### Behavioral test procedure

All behavioral sessions took place in a double-walled sound-attenuating booth. The auditory stimuli were presented diotically over Sennheiser HD 650 headphones, which have an approximately diffuse-field response; specified sound pressure levels are approximate equivalent diffuse-field levels. The tones were generated digitally and presented through a soundcard with 24-bit resolution at a sampling rate of 48 kHz. The visual stimuli were presented via a Dell 1707FPc 17” LCD monitor with a vertical refresh rate of 76 Hz placed two feet from participants’ eyes. The monitor’s luminance gamma curves were measured using a Photoresearch PR-655 and corrected in software to ensure correct display of stimulus intensity.

Participants’ thresholds were estimated using a standard two-alternative forced-choice procedure with a two-down one-up adaptive tracking rule for all three tasks [52]. For the auditory thresholds, the stimuli were always AM complex tones but either the AM or the F0 was varied depending on the task. To obtain an AM rate discrimination threshold, the F0 remained at 137 Hz but the AM rate was varied adaptively. For the F0 threshold, the AM rate remained at 13 Hz while the F0 was varied adaptively. An important point to note is that with this stimulus design, both auditory groups are exposed to the AM and the F0 of the tone but trained to discriminate only one of the two attributes. Each trial began with a 400-ms tone followed by a 200-ms gap, and then a second 400-ms tone. The background noise was gated on 500 ms before the first interval and off 200 ms after the second interval. Participants were asked to indicate via the computer keyboard which of the two tones had the higher pitch (F0) or AM modulation rate, and immediate feedback was provided after each trial.

For the visual orientation discrimination task the adaptive threshold tracked the contrast-to-noise ratio (CNR) required to discriminate the orientation of the Gabor pattern. On each trial, the Gabor pattern was presented for 200 ms followed by a 100-ms gap, and then for another 200 ms. One of the patterns was oriented at 45° and the other at 135°, randomly determined on each trial. Subjects had to indicate via button press if the second grating was rotated clockwise or counterclockwise in orientation relative to the first one.

Pre-test behavioral thresholds were obtained prior to the start of the first training session. Post-test and maintenance behavioral thresholds were obtained during the same session as their respective EEG measurements. The same adaptive procedure was implemented across the three tasks. For the auditory thresholds, the starting value of ΔAM and ΔF0 was 20%. Initially the value increased or decreased by a factor of 3. The step size was decreased to a factor of 1.41 after the first two reversals and to a factor of 1.2 after the first four reversals. For the orientation discrimination threshold, the starting value of ΔCNR was 50%. Initially the value increased or decreased by a factor of 3. The step size was decreased to a factor of 2 after the first two reversals and to a factor of 1 after the first four reversals. For all threshold measures, six reversals occurred at the smallest step size, and threshold was calculated as the geometric mean of the ΔAM, ΔF0, or ΔCNR rate value at those last six reversal points. For pre-test, post-test, and maintenance testing, each participant repeated the measures four times, and the geometric mean of the four repetitions was defined as the individual’s threshold.

### Behavioral training procedure

During each training session, participants completed 15 adaptive threshold tracks, which equates to approximately 900 trials of their designated training task. After training began, the participants were no longer exposed to the other two tasks. The adaptive tracks used for training were the same as described above in the test procedure.

### Auditory EEG analysis

EEG data were analyzed using frequency-domain principal component analysis (cPCA), as described in Bharadwaj and Shinn-Cunningham [53]. This multi-channel analysis technique allows the reduction of data acquisition time and provides a significant SNR improvement to traditional single-channel steady-state response analysis methods. The cPCA combines multichannel recordings using complex-valued weights that consider channel-specific magnitudes and phases in each frequency bin (see [53] for further details). The cPCA is also more suited for the analysis of steady-state responses in comparison to time domain analyses that combine multichannel recordings, such as principal component analysis or averaging across electrodes, because these methods assume that the signal is the same phase across sensors.

The data were first filtered into high (70–1000 Hz) and low (5–20 Hz) frequency ranges, reflecting the putative sub-cortical and cortical responses, respectively. For each filtered dataset, individual epochs were extracted beginning 50 ms before stimulus onset and extending 200 ms beyond the stimulus offset. Epochs exceeding 100 μV peak-to-peak were rejected. Visual inspection of channels with a large proportion of rejected epochs resulted in exclusion of 1–5 channels in about 1/3 of the recordings. One subject in the AM group had an unusably noisy EEG dataset from their maintenance session (i.e., a large proportion of epochs exceeding 100 μV peak-to-peak) and their maintenance data was excluded. The multi-taper complex PCA computation was completed using a single taper in both frequency ranges. Resulting phase locking values (PLV) reflect the consistency of the sustained response phase over all epochs and across all included electrodes. PLV magnitude was extracted at the experimental frequencies of the 13 Hz AM rate and the 137 Hz harmonic complex F0 in the cortical and subcortical filtered regions, respectively.

### Visual EEG analysis

Raw SSVEP data were band-pass filtered 1–59 Hz in EEGLAB [54] using a Hamming windowed sinc FIR filter, and 60-s epochs were extracted beginning at each event trigger. Epochs were transformed into the frequency domain, and signal-to-noise ratios (SNRs) were computed at expected SSVEP peaks (Gabor and noise flicker rates) compared to the average noise floor .05–0.2 Hz above and below those peaks. Occipital, parieto-occipital, and parietal electrodes (CP1, CP2, CP5, CP6, P8, P7, Pz, P3, P4, PO3, PO4, O1, Oz, and O2) were considered, and SNRs were averaged for electrodes within this set that were greater than an SNR threshold of 1.5. Five subjects had no electrodes with peaks reaching the SNR threshold in one of the three sessions (Pre-, Post- or Maintenance). For those sessions, an average of the 3 electrodes with the best SNR was used. One subject in the F0 group and one subject in the control group had unusable EEG data due to recording error and were excluded. One subject in the AM group had noisy data (i.e., a large proportion of data did not meet SNR cutoffs) only from their maintenance session so that session was excluded.

## Results

### Perceptual learning on trained and untrained tasks

**Fig 2:**
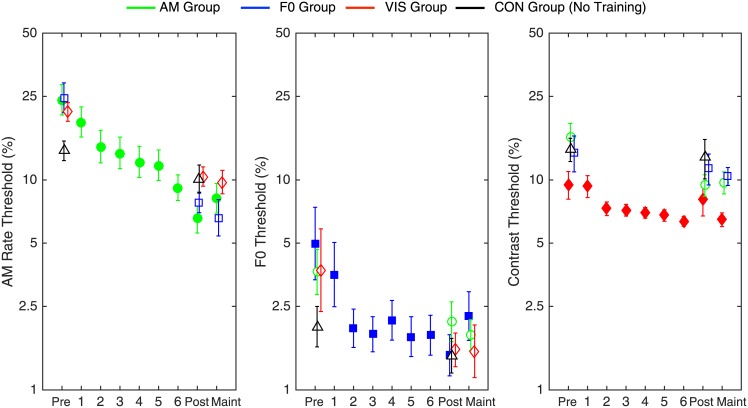
Pre-test, post-test, maintenance, and training session thresholds for the discrimination of AM rate (left), F0 (middle) and orientation (right) for the four participant groups. Participants who trained on each task are shown with filled symbols. Error bars represent ± 1 standard error of the mean.

We first assessed whether training improved behavioral discrimination thresholds. The participants trained on each task (Fig 2, filled symbols) had lower thresholds at post-test than pre-test suggesting that the training led to perceptual learning. However, improved thresholds were also seen in participants not trained on the task, including participants in the no-training CON group (Fig 2, open symbols). The thresholds were log-transformed prior to statistical analysis to maintain roughly equal variance across conditions. A Session (Pre vs. Post) by Group mixed-model ANOVA conducted for each task confirmed threshold improvements with a significant main effect of session for the AM task (*F*_1,36_ = 132, *p <* .*001*), F0 task (*F*_1,36_ = 31.7, *p <* .*001*), and the VIS task (*F*_1,36_ = 15.9, *p <* .*0001*). For both the F0 and VIS task, no significant effect of Group (F0: *F*_3,36_ = .793, *p =* .*506*, VIS: *F*_3,36_ = 1.762, *p =* .*172*) or the Session by Group interaction (F0: *F*_3,36_ = 2.255, *p =* .*099*, VIS: *F*_3,36_ = 1.502, *p =* .*230*) was observed, indicating that all groups demonstrated comparable threshold improvements.

**Fig 3:**
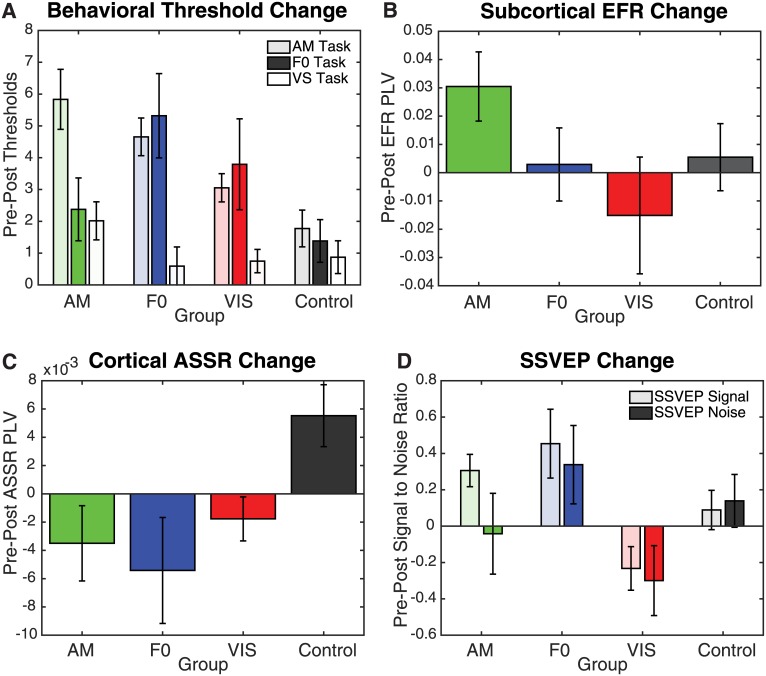
Changes in the behavioral thresholds and EEG responses from pre-test to post-test. (A) Pre—Post difference of log transformed behavioral thresholds as a function of task and training group. (B) Pre—Post difference in the PLV of subcortical EFRs as a function of training group. (C) Pre—Post difference in the PLV of cortical ASSRs as a function of training group. (D) Pre—Post difference in the SSVEP to the signal and noise as a function of training group.

For the AM task, there was a significant Session by Group interaction (*F*_3,36_ = 7.17, *p =* .*001*). Further analysis using an ANOVA with the difference between pre- and post-training threshold (Fig 3A) as the dependent variable also revealed a significant main effect of Group (*F*_3,36_ = 7.17, *p =* .*001*). Posthoc pairwise comparisons showed that the two auditory groups each had larger threshold improvements than the CON group (AM: *p <* .*001*; F0: *p =* .*004*), but there was no difference between the VIS and CON groups (*p =* .*183*). There was a significant difference between the VIS and AM group (*p =* .*033*) but no other difference between the three trained groups (*p>*.*584* in all cases).

To account for the potential effect of initial threshold variability seen in participants both within and between groups, performance on the F0 and VIS task was also converted into Pre—Post threshold difference scores (Fig 3A). However, an ANOVA with the Pre—Post threshold difference did not show a significant effect of group for either task (F0: *F*_3,36_ = 2.255, *p =* .*099*; VIS: *F*_3,36_ = 1.502, *p =* .*230)*. Surprisingly, therefore, perceptual learning was observed across all training groups with relatively little evidence of task- or even modality-specific differences.

### Neurophysiological responses

**Fig 4:**
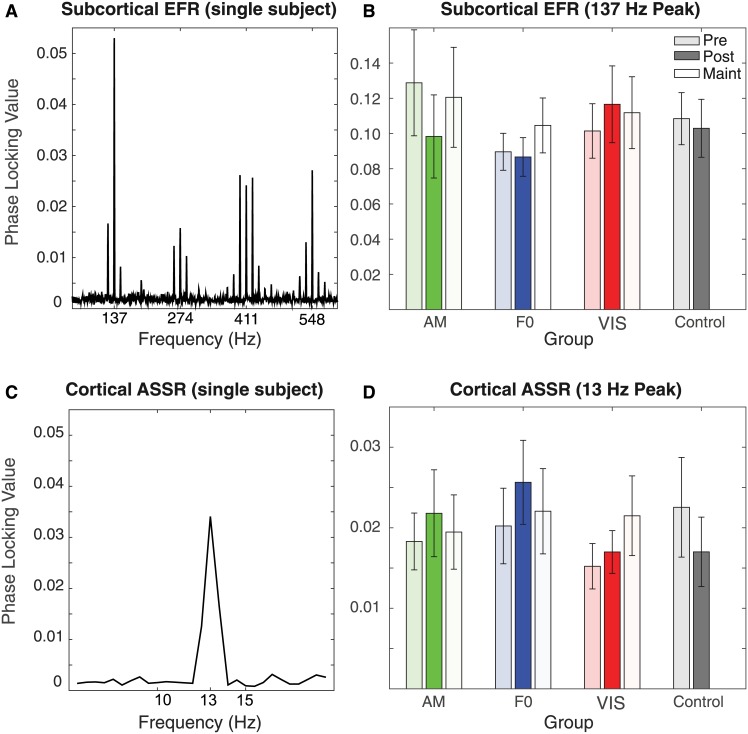
Auditory EEG results showing (A) subcortical EFR phase locking by frequency for a single, representative subject (pre-test), (B) average phase locking values at 137 Hz by group and test session, (C) cortical ASSR phase locking by frequency for the same subject as panel A, and (D) average phase locking values at 13 Hz by training group and test session. Error bars are ± 1 standard error of the mean.

To assess subcortical physiological changes in response to the training, pre- and post-test EFRs were compared (Fig 4B). A Session by Group mixed-model ANOVA on the EFR PLVs showed no significant main effect of Session (*F*_1,36_ = .637, *p =* .*430*), Group (*F*_3,36_ = .402, *p =* .*752*), or the Group by Session interaction (*F*_3,36_ = 1.591, *p =* .*209*). A second analysis was conducted to investigate the effect of group on Pre—Post EFR PLV difference (Fig 3B). An ANOVA also revealed no significant effect of group on the Pre—Post PLVs (*F*_3,36_ = 1.591, *p =* .*209*), indicating that no significant changes in the EFRs were observed after the training.

**Fig 5:**
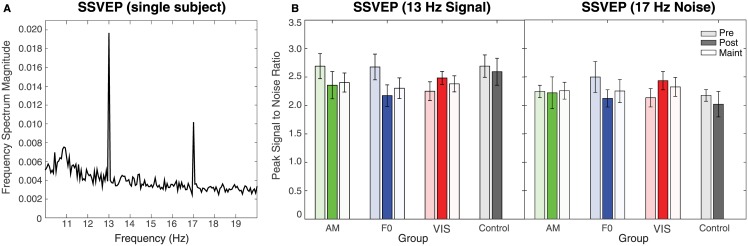
Visual EEG results showing (A) the SSVEP frequency magnitude spectrum for the subject shown in Fig 3A and 3C, and (B) group averages by test session for the 13 Hz signal (left) and 17 Hz noise flicker rates. Error bars are ± 1 standard error of the mean.

Changes in cortical responses to auditory and visual stimuli after training were assessed by comparing ASSRs (Fig 4D), SSVEP-Signal (Fig 5B, left), and SSVEP-Noise (Fig 5B, right) across pre- and post-test sessions. For the ASSRs, all three training groups showed an increase in PLV at post-test, in contrast to the CON group which showed a decrease. This pattern is captured by a significant Session by Group interaction (*F*_3,36_ = 3.23, *p =* .*034*) on a Session by Group mixed-model ANOVA. Group differences were confirmed by a significant main effect of Group (*F*_3,36_ = 3.23, *p =* .*034*) in an ANOVA with the ASSR Pre—Post Difference as the dependent variable (Fig 3C). Posthoc pairwise comparisons showed a significant difference between auditory groups and the CON group (AM: *p =* .*022*; F0: *p =* .*006*) but no significant difference between the VIS and CON groups (*p =* .*061*) or the VIS and auditory groups (AM: *p =* .*649*; F0: *p =* .*339*). This indicates that only the two auditory groups showed enhanced cortical ASSRs post-training, consistent with the pattern of AM behavioral threshold improvement.

The SSVEPs show a similar trend as the ASSRs with enhanced responses post training for the VIS group and decreased responses for the auditory and CON groups. SSVEPs to the stimuli and the noise were collapsed into a single Session (Pre vs. Post) by Group and Stimuli (Stimuli vs. Noise) mixed-model ANOVA, which revealed a significant Session by Group interaction (*F*_3,66_ = 5.50, *p =* .*002*). Further analysis with a SSVEP Pre—Post difference (Fig 3D) by Group and Stimuli ANOVA revealed group differences with a significant main effect of Group (*F*_3,66_ = 5.50, *p =* .*002*). Posthoc pairwise comparisons showed a significant difference between the VIS group and all other groups (AM: *p =* .*022*; F0: *p <* .*001*; CON: *p =* .*028*) with no other significant differences between the other groups (*p>*.*09* in all cases), suggesting that the SSVEP was enhanced only in the VIS group post training.

The correlations between behavioral threshold and EEG response difference scores were computed to assess the relationship between changes in behavioral performance and changes in the neurophysiological measures. The AM Pre—Post threshold difference was not significantly correlated with the cortical ASSR Pre—Post difference (r = -.245, *p =* .*128*). The VIS threshold difference was also not significantly correlated to the SSVEP-Signal (r = .128, *p =* .*449*) or the SSVEP-Noise Pre-Post difference (r = -.032, *p =* .*850*). Furthermore, there were no correlations between the behavioral pre-test thresholds and the EEG pre-test responses (AM Pre & ASSR Pre: r = -.257, *p =* .*109*; F0 Pre & EFR Pre: r = -.043, *p =* .*793*; VS Pre & SSVEP-Signal: r = .252, *p =* .*127*; VS Pre & SSVEP-Noise: r = .322, *p =* .*05*). Although enhancement of the cortical ASSR was seen only in the auditory trained groups and enhancement of the SSVEP was seen only in the visual trained group, there was no overall correlation between the amount of behavioral threshold improvement and change in neural response at the level of individual participants.

### Maintenance

To assess the maintenance of the enhanced cortical responses 30 days after the training, a Session (Post vs. Maintenance) by Group mixed-model ANOVA was conducted for the cortical ASSRs and the SSVEPs. No significant effect of Session (*F*_1,26_ = .108, *p =* .*745*), Group (*F*_2,26_ = .265, *p =* .*770*), or Session by Group interaction (*F*_2,26_ = 2.133, *p =* .*139*) was observed for the cortical ASSRs. Likewise, an SSVEP Session (Post vs. Maintenance) by Group and Stimuli mixed-model ANOVA revealed no significant main effect of Session (*F*_1,50_ = .086, *p =* .*770*), Group (*F*_2,50_ = .769, *p =* .*469*), or Session by Group interaction (*F*_2,50_ = .872, *p =* .*424*). Thus, no change was observed in either response one month after the training. However, another important consideration is how maintenance thresholds compare to pre-test performance. A Pre vs. Maintenance by Group mixed-model ANOVA revealed that the Session by Group interaction was no longer significant for the ASSR (*p =* .*695*) but remained significant for the SSVEP (*p =* .*002*). This outcome suggests that group differences were retained after 30 days for the SSVEP but not the ASSR.

To assess the maintenance of behavioral learning 30 days after the training, a Session (Post vs. Maintenance) by Group mixed-model ANOVA was conducted for each task. No significant effect of Session (AM: *F*_1,27_ = .239, *p =* .*629*; F0: *F*_1,27_ = 1.60, *p =* .*216*), Group (AM: *F*_2,27_ = 2.48, *p =* .*103*; F0: *F*_2,27_ = .399, *p =* .*675*), or a Session by Group interaction (AM: *F*_2,27_ = 2.85, *p =* .*076*; F0: *F*_2,27_ = 3.04, *p =* .*065*) was observed for the auditory trained groups. For the VIS task, there was no significant effect of Session (VIS *F*_1,27_ = 1.28, *p =* .*267*) or the Session by Group interaction (VIS *F*_2,27_ = .281, *p =* .*757)* but a significant effect of group *(F*_2,27_ = 4.60, *p =* .*019*), presumably reflecting the fact that the CON group appeared to have lower thresholds in both sessions on average.

## Discussion

The data presented here show enhancement of both auditory and visual cortical steady-state responses following 3 hours of training spread over 6 days. Perceptual training led to behavioral improvements in the discrimination of the F0 and AM rates of complex tones as well as the visual orientation of Gabor patterns for all training groups. Although the behavioral threshold improvements showed minimal task specificity, the neurophysiological measures more closely matched the training: auditory-trained groups demonstrated an enhancement of ASSR PLV and the visual group demonstrated an enhancement of SSVEP amplitude. Despite the fact that the modality of training and neurophysiological enhancement aligned, there was no correlation between the change in behavioral threshold and the change in physiological response. Furthermore, the VIS group showed enhancement of SSVEP magnitude to both the stimuli and the noise although they were trained only on stimuli discrimination. Similarly, enhancement of the ASSR PLV was seen in the F0 group although they were exposed to AM but not trained on AM discrimination. Finally, even though all participant groups showed improvements in the discrimination of F0, no evidence of training-induced subcortical plasticity was observed in the auditory EFR.

The results presented here demonstrate that scalp-recorded auditory and visual steady-state responses are sensitive to cortical plasticity even after very short-term perceptual learning. We have extended previous time-domain ERP results by demonstrating plasticity of steady-state responses using an analysis of PLV. Although our techniques are novel, our findings are consistent with the outcomes of other studies using different designs and measurement methods. Auditory training studies have found cortical enhancements after speech-sound training in both N1-P2 responses [30] and mismatched negativity (MMN) responses [55]. Enhancement of C1 amplitude has been documented after visual perceptual training [56], and the SSVEP has been shown to be sensitive to aversive conditioning, arguably closely related to perceptual training [44].

Our results are also consistent with a large body of animal physiological studies showing cortical plasticity following perceptual training. Recanzone et al. [57] trained monkeys on frequency discrimination and showed enhanced representation of trained frequency in A1. This finding has been replicated in other species [28,29], supporting the idea that the auditory cortex is quite malleable to experience and highlighting the importance of determining effective ways of studying human neural plasticity.

Inconsistent with our findings are previous studies that have identified enhancement of the EFR in response to short- or long-term training. Most notable is the study by Carcagno and Plack [35], who found enhanced EFR responses to both dynamic and steady pitch tokens after short-term training. One important difference between the studies may be the amount of training received by subjects. We trained subjects for a total of about 3 hours over 6 days while Cacagno and Plack’s subjects underwent a substantially greater amount of training of 10 hours over 10 days.

One surprising aspect of the visual results is the enhancement of SSVEP amplitude to both the signal and the noise. This outcome may suggest that repeated exposure to the background noise without discrimination training was sufficient to also enhance neural coding of the noise. A similar result was observed in the auditory domain with the enhancement of the ASSR PLV in the F0 group who was exposed to AM but not trained in AM discrimination. This pattern of results further suggests that stimulus exposure and procedural learning (e.g., how to direct auditory attention in the laboratory) without specific discrimination training can result in plasticity. Additional investigations are required to determine the robustness of these findings. In a study where participants were exposed to 40-Hz AM but trained only on pitch discrimination, Bosnyak et al. [58] report the opposing finding that training altered N1c and P2 evoked potentials but had no effect on the 40 Hz ASSR. One potential explanation for these inconsistent findings is that sustained cortical potentials in response to slower modulation rates may be more plastic [48].

Directed attention has been shown to modulate the ASSR amplitude both for the lower speech-related AM rates we used [48] and for more rapid rates [59,60]. It may be that the mechanisms of short-term learning observed here are more closely related to the mechanisms of directed sensory attention than to the longer-term mechanisms underlying the enhancement of musicians’ and tonal language speakers’ EFRs [61]. Such long-term training is complicated by personal, social, and emotional factors that are hard to replicate in the laboratory but may heighten the importance of certain sounds and the responses they elicit. Polley et al. [62] provide evidence suggesting that top-down, task-dependent factors, arising from multiple cortical areas play a role in the nature of observed physiological changes. While past studies [48] have reported the effect of attention on the amplitude of the ASSR, there is much less evidence for the effect of attention on measures of the ASSR’s PLV. If the ASSR change observed here is indeed related to the direction of attention, our findings suggest that PLV holds potential as a tool for studying the effects of attention on neurophysiological responses.

Although the results of human EEG studies show enhancement of neurophysiological responses to trained stimuli, we cannot determine the exact neural mechanisms underlying the modulation of measured responses. It may be that training increases the synchrony (phase locking) of neural fibers to the AM of the visual or auditory stimulus or that an increased number of fibers are recruited to the processing of a trained stimulus. Combining behavioral and human results like these with modeling of the auditory and visual pathways and ongoing animal work may help to unravel the question of what neuronal response properties are causally related to perceptual improvements. An important limitation to note is that the actual sources of the EEG signals were not localized in this study. There is a general consensus that the neural sources of the fast-rate EFR are primarily subcortical and the sources of the slow-rate ASSR are primarily cortical [14,15,53]. However, a recent study that utilized magnetoencephalography for source localization, showed a right hemisphere cortical contribution in addition to the subcortical sources for the EFR [63]. Our use of cortical versus subcortical with reference to the primary generators of the ASSR and EFR, though consistent with past literature, is certainly an oversimplified consideration of their neural sources. Furthermore, it is also possible that a common neural rate-discrimination mechanism could account for both high-rate (F0) and low-rate (AM) discrimination, regardless of where the responses are generated. This could serve as an alternate explanation for the increase in ASSR PLV observed in the F0 group who was trained on high-rate discrimination but showed low-rate physiological enhancement. Additional investigation into the generalization of different high and low training rates is required to confirm this possibility.

Although the main question addressed in this study was whether there was evidence of plasticity in the steady-state EEG responses, the perceptual training paradigm produced an interesting and complex pattern of behavioral results. We found threshold improvements on all three tasks at post-test for all groups including the control group. Improvement in the no-training control group is actually a common phenomenon (for example, see [64]). However, this finding also speaks to the effect of stimulus exposure on behavioral measures of learning. Participants in our study were exposed to the stimuli significantly more than is typical in a behavior-only training paradigm with the one thousand stimulus repetitions presented during the EEG recording. Procedural learning, or the impact of subjects acclimating to the environment and nature of psychophysical tasks, is also likely to have impacted our behavioral results, as all of our subjects were entirely naïve to psychophysical experiments before participating in this study. Although these effects have been demonstrated before, our data show a potentially large effect of these factors on the differences between pre-test and post-test thresholds.

Our behavioral findings also provide evidence for cross-modal transfer of learning. Participants trained on the auditory tasks showed improvement on the visual threshold while those trained on the visual task showed improvement on the auditory thresholds. This type of cross-modal effect was not seen in the EEG data (i.e., subjects learned behaviorally across modalities but did not exhibit any changes in cross-modal physiological responses). Neurophysiological enhancement matched the modality of training. One way to interpret this pattern of results is that the cross-modal transfer of behavioral learning may have occurred at a later stage, such as decision formation [65,66], and therefore did not affect stimulus coding as measured by EEG. It is important to note, however, that one complication in the interpretation of our behavioral results is the large difference in mean thresholds between the groups at pre-test. Additional analysis of the Pre—Post threshold difference scores to account for initial baseline variability, nevertheless, did not change the results.

In conclusion, this study provides evidence that short-term, learning-related physiological changes may be measured in the adult auditory and visual cortex using EEG. Simultaneous subcortical and cortical sustained responses provide a unique insight into how different levels of the auditory pathway respond to amplitude modulation and how those responses might be sensitive to perceptual learning. Our findings suggest that cortical responses may be more reflective of training-induced plasticity than subcortical responses and that modality-based specificity is more apparent than task-based specificity. In contrast, our behavioral results revealed apparent cross-task and cross-modality transfer of learning.
